# Changes in Macular Microvascular Structure in Macular Edema Secondary to Branch Retinal Vein Occlusion Treated with Antivascular Endothelial Growth Factor for One Year

**DOI:** 10.1155/2021/6645452

**Published:** 2021-05-17

**Authors:** Shuang Song, Xiaobing Yu, Peng Zhang, Hong Dai

**Affiliations:** Department of Ophthalmology, Beijing Hospital, National Center of Gerontology, Institute of Geriatric Medicine, Chinese Academy of Medical Sciences, Beijing, China

## Abstract

**Purpose:**

To observe the changes in macular microvascular structure and the correlation between anatomy and visual function in patients with macular edema secondary to branch retinal vein occlusion (BRVO) treated with antivascular endothelial growth factor for one year.

**Methods:**

This prospective study enrolled 39 patients (one eye per patient) who received intravitreal injections of ranibizumab for macular edema secondary to BRVO. All patients received a minimum of 3 initial monthly ranibizumab injections and criteria-driven pro re nata (PRN) dosing thereafter for visual acuity (VA) and central retinal thickness (CRT) stabilization. The follow-up period of this study was one year. The vascular density (VD) of the superficial retinal capillary plexus (SCP) and deep retinal capillary plexus (DCP), the foveal avascular zone (FAZ) area, the FAZ perimeter, the VD within a 300 *μ*m wide ring surrounding the FAZ (FD-300), and the acircularity index (AI) were measured automatically by optical coherence tomography angiography (OCTA) at baseline, month 6, and month 12.

**Results:**

Compared with those before treatment, the VD of the SCP significantly decreased 6 months after treatment (*P* < 0.05), while the area and perimeter of the FAZ increased significantly (*P* < 0.01). After 12 months of treatment, the area and perimeter of the FAZ increased significantly (*P* < 0.01). There was no significant difference in any parameters between 12 months and 6 months after treatment (*P* > 0.05). The change in BCVA was negatively correlated with the VD of the SCP at 12 months (*P*=0.0447, *r* = −0.3233). There was a relationship between the DBP and AI, and CRT was related to VD of DCP at baseline (*P*=0.028,  0.0209; *r* = 0.383, −0.384). The PERIM and AI at 12 months were significantly associated with the recurrence of macular edema, and the changes in vascular density in the SCP and PERIM were significantly associated with the number of injections within 12 months (*P* < 0.05).

**Conclusions:**

One year after ranibizumab treatment, the area and perimeter of the FAZ were enlarged, while the VD of the SCP and DCP remained stable, which indicated that ranibizumab treatment did not improve macular blood supply and macular ischemia in BRVO patients.

## 1. Introduction

Branch retinal vein occlusion (BRVO) is a common sight-threatening retinal vascular disease. The prevalence of BRVO is 4.42 cases per 1,000 people [1, 2]. Macular edema (ME) secondary to BRVO is considered the main cause of visual impairment [3]. At present, the treatment options for BRVO include antivascular endothelial growth factor (VEGF), corticosteroids, and macular lasers [4, 5].

Recently, the application of optical coherence tomography angiography (OCTA) has renovated our approach to study the pathology of retinal vessels and given us a new view for deep analysis of the involved structures. OCTA, as a novel dyeless technology, is instantaneous, fast, noninvasive, and high resolution, and it clearly shows retinal vessels of different levels and enables a direct view of the retinal capillary plexus (DCP), which is involved in BRVO and is not visible on fluorescein fundus angiography (FFA); additionally, the observation of abnormal vessels is clearer than in traditional fluorescein fundus angiography (FFA) [6]. The retinal vascular density (VD) and the foveal avascular zone (FAZ) of the macular fovea are measured quantitatively for different layers [7, 8]. Several studies have indicated that OCTA can be used to evaluate microvascular changes in patients with BRVO before and after anti-VEGF treatment and as a valuable imaging tool for the follow-up evaluation in BRVO eyes [7–11]. However, a few studies have reported the correlation of anatomy and visional function in BRVO eyes evaluated with OCTA.

To evaluate the changes in macular microvascular structure with ranibizumab intravitreal treatment and the correlation of anatomy and visional function in BRVO eyes, we conducted this study of monocular BRVO patients who were examined with OCTA.

## 2. Materials and Methods

### 2.1. Subjects

This was a prospective study that followed the principles of the Declaration of Helsinki and was approved by the Ethics Committee of Beijing Hospital. The study was conducted between February 2017 and August 2019. All participants signed a standard informed consent form which included information on the potential risks and benefits of the procedure and subsequent management; they could not be identified through this document. We followed the methods of Song et al. [12].

This study included 39 patients (one eye per patient) who were ultimately diagnosed with ME due to BRVO between February 2017 and August 2019. All patients were confirmed by the ophthalmology department of Beijing Hospital with a comprehensive examination including blood pressure, best-corrected visual acuity (BCVA), intraocular pressure (IOP), slit-lamp biomicroscopy, autorefractometry, gonioscopy, OCTA, fluorescein fundus angiography (FFA), and dilated fundoscopic examinations of both eyes.

The study enrolled treatment-naïve patients older than 18 years who had suffered from ME secondary to BRVO within 12 months. The BCVA letter score at baseline was between 24 and 73 Early Treatment Diabetic Retinopathy Study (ETDRS) letters (approximate Snellen chart equivalent of 20/400 and 20/40), and the central retinal thickness (CRT) was more than 250 *μ*m. Patients were excluded if they met the following criteria: (1) hemi-CRVO or CRVO; (2) diabetic maculopathy and/or retinopathy; (3) any other BCVA compromising ocular disease; (4) any prior intravitreal anti-VEGF or corticosteroid injections; (5) any prior retinal laser photocoagulation; (6) IOP higher than 21 mmHg; (7) history of vitrectomy; (8) history of myocardial infarction or stroke within three months; and (9) other major systemic disorders.

### 2.2. Treatments

According to the treatment protocol, all patients received 3 initial intravitreal ranibizumab (IVR) monthly treatments, which were repeated as needed (3 + PRN). BCVA and CRT were the primary triggers of retreatment. The patients and the investigators responsible for the treatment were both masked, and the decision for retreatment was based on the changes in BCVA and CRT. Eyes with more than 5 letters (ETDRS) loss due to disease activity or a more than 100 *μ*m increase in CRT received retreatment every 4 weeks.

Ranibizumab was administered at a dose of 0.5 mg/0.05 ml per injection. After instilling topical 0.4% oxybuprocaine chloride eye drops for topical anesthesia, the eye was irrigated with 5% povidone-iodine and opened using a lid retractor, and the drug was injected through the pars plana 3.5 mm posterior to the limbus in the inferotemporal quadrant in pseudophakic eyes and 4.0 mm posterior to the limbus in phakic eyes using a 30 G needle. The treatment protocol was similar to Gu's study [13].

All patients who met the retreatment criteria received reinjection of ranibizumab during the follow-up period.

### 2.3. Best-Corrected Vision Acuity

The BCVA of patients was assessed with ETDRS VA testing charts by a certified examiner at baseline and at every follow-up visit. The standard testing distance was 4 meters, and it was changed to 1 meter if a patient could not read at least 4 letters at 4 meters [14].

### 2.4. Optical Coherence Tomography Angiography (OCTA)

OCTA examinations were conducted to measure macular vessel density and CRT with automated measurements provided by spectral-domain OCT (RTVue XR Avanti, Optovue, Inc., Fremont, CA, USA) and AngioVue software (version 2017.1.0.151, Optovue, Inc.) during every visit.

We focused on a 3 × 3 mm scan area centered on the fovea to quantify OCTA parameters by 304 × 304 B-scan lines for all patients. The wavelength of the light source was 840 nm, and the bandwidth was 45 nm. The scanning frequency of A-scan was 70000 Hz. Single OCTA image acquisition included one horizontal scan and one vertical scan to avoid artifacts of eye movement.

We only chose OCTA images with an image quality score of 6 or more. The VD of the superficial retinal capillary plexus (SCP), DCP, FAZ area, perimeter (PERIM), VD within the range of 300 *μ*m, acircularity index (AI), FAZ, and foveal central retinal thickness (CRT) were measured automatically by OCTA built-in software on the foveal area. Based on the default setting of the OCTA system, the SCP of the retina included blood vessels from the internal limiting membrane (ILM) to –10 *μ*m below the inner plexiform layer (IPL). The DCP of the retina included blood vessels from –10 *μ*m below the IPL to 10 *μ*m below the outer plexiform layer (OPL). The FAZ was defined as a capillary-free area in the central macular region on traditional FFA analysis of the retina. Using a retinal angiography scan, the latest FAZ measurements were obtained based on the retina slab from the ILM to 10 *μ*m below the OPL. This measurement provided the following parameters: the FAZ area; the PERIM; the AI, which is defined as the ratio of the perimeter of the FAZ and the perimeter of a circle with equal area, AI = PERIM/equal area standard circumference; and the FD-300, which refers to the blood vessel density within a 300 *μ*m wide ring around the FAZ. The FAZ area, PERIM, AI, and FD-300 were used to evaluate the hemodynamics of the FAZ.

The VD of SCP and DCP and FAZ parameters in the central macular area of eyes with BRVO before and after treatment with anti-VEGF drugs and their relationship with BCVA were observed.

### 2.5. Efficacy and Safety Assessments

The incidence of ocular or nonocular adverse events (AEs) and severe adverse events (SAEs) was assessed during the follow-up period.

### 2.6. Statistical Analysis

Statistical analyses were performed with SAS 9.4 (SAS Institute Inc. Cary, North Carolina, USA), and statistical significance was established at two-tailed *P* < 0.05. Data were summarized as numbers (percentages), means ± standard deviations (SDs), or medians (interquartile ranges (IQRs)) as appropriate. Student's paired *t*-test and Wilcoxon test were adopted to compare changes before and after ranibizumab treatment in BRVO eyes. To analyze changes in BCVA, as well as CRT, general linear models were constructed for variance analysis with baseline BCVA/CRT adjusted. Logistic regression analyses were adopted to analyze the correlation between the changes in the evaluated parameters for variables and recurrence of macular edema or the number of injections. Safety analyses were conducted on the safety set. Adverse events (AEs) were summarized by reporting the number and percentage of patients with any ocular and/or nonocular AEs.

## 3. Results

In this study, we evaluated 39 eyes from 39 patients with ME due to BRVO, including 28 males and 11 females. The mean age of the selected patients was 58.8 ± 10.8 years, the mean SBP was 134.6 ± 11.4 mmHg, and the mean DBP was 81.5 ± 9.9 mmHg. The mean IOP was 15.6 ± 2.5 mmHg. Sixteen patients received intravitreal ranibizumab 0.5 mg alone, and 23 patients received intravitreal ranibizumab 0.5 mg with grid laser photocoagulation (Table 1).

### 3.1. Best-Corrected Visual Acuity

At baseline, the mean BCVA was 56.5 ± 9.4 letters based on the ETDRS chart. After ranibizumab treatment, the mean BCVA was 72.4 ± 10.6 letters at the last visit, which was significantly improved compared to the baseline BCVA (*P* < 0.001) (Tables 1 and 2).

### 3.2. Central Retinal Thickness

The mean CRT was 538.2 ± 192.9 *μ*m at the first visit. The CRT was reduced significantly to 248.6 ± 93.6 *μ*m at the last visit after the ranibizumab injection (*P* < 0.001) (Tables 1 and 2).

### 3.3. Foveal Avascular Zone

**Figure 1:**
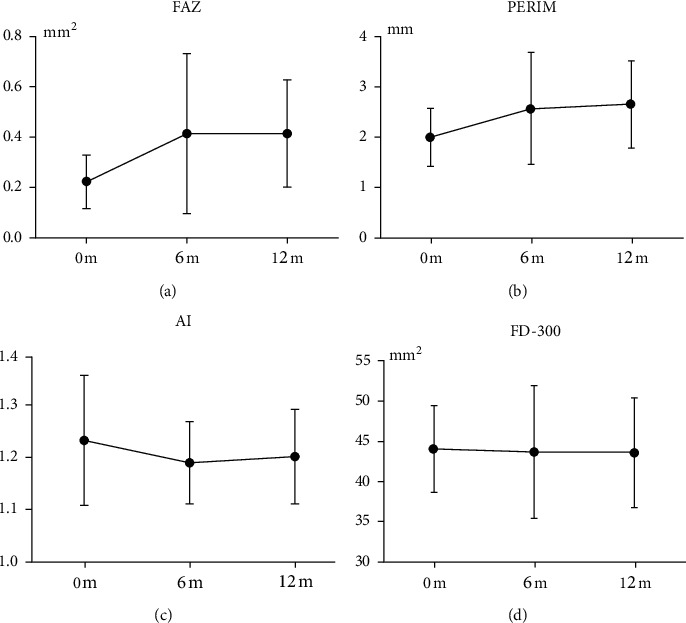
Changes in FAZ, PERIM, AI, and FD-300 within 12 months. FAZ, foveal avascular zone; PERIM, FAZ perimeter; AI, acircularity index; FD-300, the VD within a 300 *μ*m wide ring surrounding the FAZ.

The FAZ parameters, including the FAZ area, PERIM, AI, and FD-300, are shown in Table 2. After ranibizumab treatment, the enlargement of the FAZ area and the increase in PERIM were significant (*P* < 0.01) at both month 6 and month 12. However, there were no significant differences in FD-300 and AI (*P* > 0.05) at either month 6 or month 12. The changes in FAZ, PERIM, AI, and FD-300 within 12 months are shown in Figure 1.

### 3.4. Macular Vascular Density

**Figure 2:**
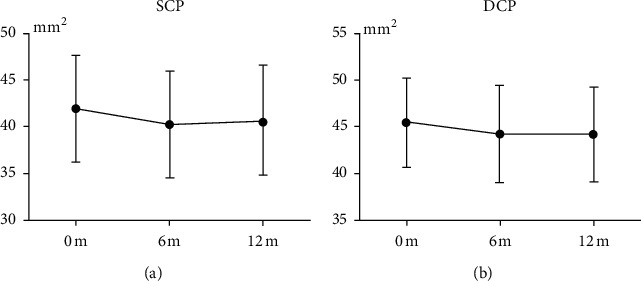
Changes in macular vascular density of the SCP and DCP within 12 months. SCP, superficial retinal capillary plexus; DCP, deep retinal capillary plexus.

The vascular density measurements of the fovea and separate sectors of parafovea of the SCP and DCP are shown in Table 3. After 6 months, VD of SCP decreased significantly (*P*=0.041), but there were no significant differences in DCP (*P* > 0.05). After 12 months, there were no significant differences in SCP and DCP (*P* > 0.05). The changes in the macular vascular density of the SCP and DCP within 12 months are shown in Figure 2.

### 3.5. Correlation Analysis between Different Parameters

Pearson correlation analysis between the BCVA and OCTA parameters was conducted in our study, and the significant findings are shown in Tables 4 and 5. We also conducted a correlation analysis between CRT and OCTA parameters in patients with BRVO.

There was a relationship between DBP and AI and CRT related to DCP at baseline (*P*=0.028,  0.0209; *r* = 0.383, −0.384). BCVA was negatively correlated with the macular vascular density of SCP at month 12 (*P*=0.0447, *r* = −0.3233).

### 3.6. Correlation Analysis between the Recurrence of Macular Edema and the Number of Injections with the Evaluated Parameters

The recurrence of macular edema was considered to have occurred when macular edema disappeared with CRT <300 *µ*m after injection of ranibizumab at month 6, but macular edema recurred with CRT ≥300 *µ*m at month 12. The rate of recurrence of macular edema was 5/39 in this study. The different parameters evaluated by OCTA were grouped by whether they increased or decreased based on the changes in these parameters from baseline to month 12. Binary logistic regression analysis was adopted to analyze the correlation between the recurrence of macular edema and the evaluated parameters (Table 6). Linear logistic regression analyses were adopted to analyze the correlation between the number of injections and the evaluated parameters (Table 7). The PERIM and AI at month 12 were significantly associated with the recurrence of macular edema (Table 6). The PERIM was negatively correlated with the recurrence of macular edema, but the AI was positively correlated with the recurrence of macular edema at month 12 (Table 6). The changes in vascular density in the SCP and PERIM were significantly associated with the number of injections at month 12 (Table 7). The increases in vascular density in the SCP and PERIM were both negatively correlated with the number of injections within 12 months (Table 7).

### 3.7. Adverse Events

Two patients in the IVR group exhibited an IOP increase during the study. IOP return to normal levels with topical therapy. No other AEs or SAEs of any kind were recorded during the study period.

## 4. Discussion

In this study, we found that the visual acuity of BRVO patients significantly improved and CRT significantly decreased, but the area and perimeter of the FAZ were enlarged, and the VD of macular capillaries was not significantly improved one year after treatment with ranibizumab, as detected by OCTA software. These results indicated that although intravitreal injection with ranibizumab can effectively treat macular edema, it cannot improve macular blood supply and macular ischemia in patients with BRVO. Our previous studies showed that there was no significant change in macular ischemia before and after treatment with ranibizumab for one month. However, macular ischemia was gradually aggravated after 3 months of this treatment similar to the results of Deng's study [8]. In this study, we found that macular ischemia was aggravated within 6 months after treatment with ranibizumab, and macular ischemia persisted from 6 months to one year and was relatively stable and did not improve or aggravate. Feucht found that FAZ increased in BRVO patients treated with anti-VEGF drugs for 2 months by FFA examination [15], which was the same as the OCTA examination results. Some studies have proposed that anti-VEGF drugs lead to transient contraction of retinal arteriovenous vessels, which leads to a decrease in blood density and slight expansion of the FAZ [16]. Some studies have also suggested that if blood flow is below the limitation of 0.3 mm/s, it will not be detected by OCTA, which may be one of the reasons for the enlargement of the FAZ [9]. Campochiaro found that the retinal nonperfusion area appeared in both the BRVO anti-VEGF treatment group and the nontreatment group after one year by FFA examination, but the retinal nonperfusion area appeared more quickly in the nontreatment group; the anti-VEGF therapy is considered to reduce the progression of the retinal nonperfusion area but not completely prevent the occurrence of nonperfusion area [17]. We speculated that the aggravation of macular ischemia in patients with BRVO within one year may be the natural progression of this disease, and anti-VEGF drugs may only delay the progression of macular ischemia and not completely prevent it.

Different effects of anti-VEGF drugs on retinal vascular density in patients with RVO have been reported recently. Some studies have shown that macular vascular density increases after anti-VEGF treatment in RVO [11, 18, 19]. This may be due to the decrease in VD caused by acute macular edema, which can be improved with reduced macular edema after anti-VEGF treatment [11, 18]. Other authors suggested that anti-VEGF drugs stimulated the production of NO to cause vasodilation, which results in an increase in VD [19]. Similar to the results of this study, Spaid found that anti-VEGF treatment of BRVO could not improve macular vascular density and macular ischemia [20]. In this study, we found that 6 months after treatment with ranibizumab, the VD of SCP decreased significantly, while the VD of DCP did not decrease significantly. It is speculated that the decrease in VD in SCP may be due to tissue edema caused by obstruction of superficial retinal vascular reflux during RVO, which causes vascular occlusion of SCP for a long time. Even after anti-VEGF treatment, the probability of recanalization of SCP blood vessels is relatively low, so the VD of SCP decreases. We speculated that macular edema was mostly located in the deep layer, macular edema decreased, and deep retinal capillaries were rearranged after 6 months of anti-VEGF treatment, resulting in no obvious change in VD of the DCP. Most previous studies report results a short time (1–6 months) after anti-VEGF treatment [10, 15, 16, 21]. This study focused on observing the ischemic process for one year after anti-VEGF treatment, and the observation time was much longer. The results of our study 6 months after anti-VEGF treatment also showed that there was no significant change in VD between 6 months to 1 year after treatment and 1 year after treatment. These results indicated that VD was relatively stable for 6 months and the whole year after treatment. The VD of SCP decreased at 6 months, but it increased slightly at one year. Therefore, there was no significant change in the VD of the SCP at the end of one year. To the best of our knowledge, VD changes in SCP within 1 year after anti-VEGF treatment have not been previously reported.

We also found that BCVA in patients with BRVO was negatively correlated with the VD of SCP but was not significantly correlated with that of DCP after 12 months of treatment with ranibizumab. The visual acuity of BRVO patients was significantly improved, and CRT decreased significantly after one year of treatment with ranibizumab. This outcome may be due to the reduced blood supply of superficial vessels in BRVO patients, which leads to ischemia and hypoxia of superficial retinal tissue and atrophy in the superficial retina. We also found that macular edema recurred less frequently in eyes with retinal atrophy, which retained better visual function. Therefore, we speculated that the lower the VD of the SCP at 12 months, the better the visual acuity. There has been no recent report on the correlation between BCVA and SCP for one year, and the specific mechanism needs to be further studied.

We also found that macular edema (CRT) was negatively correlated with the VD of the DCP at baseline but not that of the SCP. Moussa indicated that CRT was associated with ischemia of DCP [22]. The macular area is mainly the outer structure of the retina, which is supplied by the choroid. Retinal vein ischemia has a significant effect on the VD of the DCP in patients. The decrease in VD in the DCP leads to a decrease in retinal interstitial fluid clearance, resulting in macular edema [23]. Therefore, the reduction in VD of the DCP is thought to be closely related to the increased CRT. One study showed that it was difficult for occluded vessels to be completely reperfused even after anti-VEGF therapy [18]. Based on the results of our study, we speculate that deep blood flow obstruction is more serious in patients with BRVO, leading to more significant macular edema.

There was no significant correlation between FAZ and visual acuity in this study. Many studies have previously reported a correlation between vision and FAZ, but the conclusions have been inconsistent [6, 24–26]. Many previous studies have found that enlargement of the FAZ was related to reduced visual acuity [25, 26]. Sophie found a negative correlation between the nonperfusion area of the macular zone and the change in visual acuity in patients with BRVO after treatment with ranibizumab [24]. Farinha found that a larger FAZ and poorer baseline BCVA led to worse visual function in the ischemia group [27]. Samara found that the FAZ was significantly enlarged in the DCP, which was positively correlated with visual acuity [25]. In this study, we found that there was no significant correlation between FAZ and visual acuity, which was inconsistent with the results of previous studies; this discrepancy may be related to the smaller sample size.

In this study, we found that the PERIM was negatively correlated with the recurrence of macular edema, but the AI was positively correlated with the recurrence of macular edema at month 12. The increases in vascular density in both the SCP and PERIM were negatively correlated with the number of ranibizumab injections within 12 months. Tomita indicated that eyes with a greater loss of macular capillaries were more likely to have fewer recurrences of macular edema and fewer injections within 6 months [28], and Choi found that nonperfusion status in SCP or DCP was strongly correlated with ME recurrence within 6 months [29]. In this study, we analyzed the changes in the evaluated parameters within 12 months, which was a much longer period. From this study, we found that eyes with an increase in vascular density in SCP received fewer injections of ranibizumab within 12 months. We suggest that eyes with an increase in vascular density in SCP may have improved oxygen levels, which decrease VEGF production, leading to fewer injections. There was no significant difference between the vascular density in SCP at baseline and recurrences of macular edema or the number of injections within 12 months. We found that eyes with a larger perimeter of the FAZ were more likely to have fewer recurrences of macular edema at month 12, and eyes with an increase in the perimeter of the FAZ were more likely to receive fewer injections within 12 months. The possible mechanism is that eyes with a greater FAZ may have atrophy of the inner retinal layer, lower oxygen demand, and lower VEGF production, leading to fewer recurrences of macular edema and fewer injections. There have been a few recent studies on the significance between AI and the number of injections, and larger studies need to be conducted.

Furthermore, we found that diastolic blood pressure was related to AI. Hypertension is one of the risk factors for BRVO. Increased diastolic blood pressure can lead to an abnormal vascular microenvironment, vascular hardening, and eventually vascular obstruction. Deng declared that compared with the normal control group, the AI of eyes with BRVO was higher [8]. There have been a few studies on the significance of AI, and the specific mechanism of the relationship between diastolic blood pressure and AI needs to be further studied.

The limitations of our study include the small sample size, which was not sufficient to draw definitive conclusions. Second, the OCTA images we obtained only focused on the foveal area, and the region affected in BRVO eyes was usually occupied outside the 3 × 3 mm OCTA images. We will conduct further studies to investigate vessel density changes in the peripheral fundus region. Third, patients who obtained good fixation were enrolled in this study to obtain clear flow maps in OCTA. Therefore, some patients with BRVO were excluded for poorer vision. In addition, the vessel density data of the fellow eye in BRVO patients were not analyzed, and healthy controls were missing. We did not evaluate other disease-induced anatomic changes, such as ellipsoid zone disruption and intraretinal cysts, which may have also influenced VA.

## 5. Conclusions

The current study suggested that one year after ranibizumab treatment, the area and perimeter of the FAZ were enlarged, while the VD of the SCP and DCP remained stable, which indicated that ranibizumab treatment could not improve macular blood supply and macular ischemia in BRVO patients. This study also indicated that CRT was related to DCP at baseline, and the macular vascular density in the SCP decreased significantly at month 6. The FAZ and FAZ perimeter increased significantly at month 6 and month 12. The changes in BCVA were negatively correlated with macular vascular density in SCP at month 12. The PERIM was negatively correlated with the recurrence of macular edema, but the AI was positively correlated with the recurrence of macular edema at month 12. The increases in vascular density in both the SCP and PERIM were negatively correlated with the number of injections within 12 months. Our study suggests the potential contribution of OCTA as a novel noninvasive imaging technology that enables qualitative and quantitative monitoring of macular edema in BRVO and may be a preferred tool for the diagnosis and longitudinal evaluation of BRVO. Further studies with larger sample size and longer follow-up periods should be further explored to evaluate the reliability of these findings.

## Figures and Tables

**Table 1:** Baseline demographics and ocular characteristics of participants.

Variables	
Age (years)	58.77 ± 10.88
Gender (male)	28 (71.8%)
SBP (mmHg)	134.6 ± 11.4
DBP (mmHg)	81.5 ± 9.9
BCVA	56.5 ± 9.4
CRT (*μ*m)	538.2 ± 192.9
Intraocular pressure	15.6 ± 2.5
SCP (mm^2^)	41.91 ± 5.70
DCP (mm^2^)	45.49 ± 4.74
FAZ area (mm^2^)	0.22 ± 0.11
Perimeter (mm)	2.01 ± 0.57
Acircularity index	1.23 ± 0.12
FD 300 area density (mm^2^)	44.03 ± 5.37

SBP, systolic blood pressure; DBP, diastolic blood pressure; BCVA, best-corrected visual acuity; CRT, central retinal thickness; SCP, superficial retinal capillary plexus; DCP, deep retinal capillary plexus; FAZ, foveal avascular zone.

**Table 2:** The data of participants at the last visit.

Variables	
Injection numbers	5.0 ± 2.0
SBP (mmHg)	135.8 ± 12.8
DBP (mmHg)	80.6 ± 10.4
BCVA	72.4 ± 10.6
CRT (*μ*m)	248.6 ± 93.6
Intraocular pressure (mmHg)	15.4 ± 3.5
SCP (mm^2^)	40.7 ± 5.9
DCP (mm^2^)	44.2 ± 5.0
FAZ area (mm^2^)	0.41 ± 0.21
Perimeter (mm)	2.67 ± 0.86
Acircularity index	1.20 ± 0.09
FD 300 area density (mm^2^)	43.6 ± 6.81

SBP, systolic blood pressure; DBP, diastolic blood pressure; BCVA, best-corrected visual acuity; CRT, central retinal thickness; SCP, superficial retinal capillary plexus; DCP, deep retinal capillary plexus; FAZ, foveal avascular zone.

**Table 3:** Differences in macular vascular density at month 6 and month 12 from baseline.

Characteristics	No.	Month		After-before difference between months 95% CI	*P* value
		After	Before		
SCP	39	6	0	−1.654 (−3.237, −0.071	0.0411^*∗*^
		12	0	−1.139 (−2.652, 0.375)	0.1361
		12	6	0.515 (−0.750, 1.781)	0.4148

DCP	39	6	0	−1.095 (−3.088, 0.899)	0.2732
		12	0	−1.219 (−3.198, 0.760)	0.2199
		12	6	−0.125 (−1.577, 1.327)	0.8630

FAZ area	39	6	0	0.200 (0.100, 0.300)	0.0002^*∗*^
		12	0	0.193 (0.124, 0.262)	<0.0001^*∗*^
		12	6	−0.007 (−0.083, 0.068)	0.8446

Perimeter	39	6	0	0.604 (0.238, 0.970)	0.0019^*∗*^
		12	0	0.669 (0.375, 0.962)	<0.0001^*∗*^
		12	6	0.065 (−0.227, 0.358)	0.6544

Acircularity index	39	6	0	−0.043 (−0.096, 0.010)	0.1064
		12	0	−0.031 (−0.080, 0.018)	0.2039
		12	6	0.012 (−0.017, 0.041)	0.3972

FD-300 area density	39	6	0	−0.220 (−2.938, 2.498)	0.8707
		12	0	−0.318 (−2.624, 1.987)	0.7814
		12	6	−0.098 (−2.475, 2.278)	0.9337

SCP, superficial retinal capillary plexus; DCP, deep retinal capillary plexus; FAZ, foveal avascular zone. ^*∗*^*P* < 0.05.

**Table 4:** Correlations between BCVA, CRT, intraocular pressure, SBP, DBP, and indexes of macular vascular density at baseline.

Characteristics	Correlation	SCP	DCP^†^	FAZ area	Perimeter	Acircularity index^†^	FD-300 area density
SBP	N	36	36	33	33	33	33
	*P* value	0.9	0.6389	0.6680	0.6324	0.3818	0.6785
	r coefficient	−0.022	0.081	−0.079	−0.086	0.157	−0.075

DBP	N	36	36	33	33	33	33
	*P* value	0.8758	0.8073	0.4193	0.223	0.028^*∗*^	0.9256
	r coefficient	−0.027	−0.042	0.145	0.218	0.383	0.017

BCVA	N	36	36	33	33	33	33
	*P* value	0.4277	0.168	0.6661	0.5602	0.5857	0.8104
	r coefficient	−0.136	0.235	0.078	0.105	0.098	−0.043

CRT^†^	N	36	36	33	33	33	33
	*P* value	0.844	0.0209^*∗*^	0.9713	0.8716	0.4666	0.0838
	r coefficient	−0.034	−0.384	−0.007	−0.029	−0.131	0.306

Intraocular pressure	N	36	36	33	33	33	33
	*P* value	0.5418	0.8361	0.9474	0.8722	0.2935	0.5821
	r coefficient	−0.105	0.036	−0.012	−0.029	0.188	−0.099

SBP, systolic blood pressure; DBP, diastolic blood pressure; BCVA, best-corrected visual acuity; CRT, central retinal thickness. ^†^At baseline, CRT, DCP, and acircularity index were abnormally distributed (*P*=0.0473,  0.0001,  and 0.0006, respectively, in Shapiro–Wilk's test). The Spearman correlation was used to examine correlations with other variables. Pearson correlations were analyzed for others. ^*∗*^*P* < 0.05.

**Table 5:** Correlations between BCVA, CRT, intraocular pressure, SBP, DBP, and indexes of macular vascular density at month 12.

Characteristics	Correlation	SCP	DCP	FAZ area	Perimeter	Acircularity index^†^	FD-300 area density
SBP	N	39	39	39	39	39	39
	*P* value	0.8277	0.2257	0.4059	0.2163	0.078	0.0682
	r coefficient	0.036	0.1985	0.1369	0.2025	0.2856	0.2951

DBP	N	39	39	39	39	39	39
	*P* value	0.8525	0.7325	0.7631	0.5917	0.9188	0.7304
	r coefficient	−0.0308	−0.0565	−0.0499	−0.0866	−0.0169	−0.057

BCVA	N	39	39	39	39	39	39
	*P* value	0.0477^*∗*^	0.334	0.9483	0.9983	0.5116	0.0957
	r coefficient	−0.3233	−0.1589	−0.0107	0.0004	0.1083	−0.2706

CRT^†^	N	39	39	39	39	39	39
	*P* value	0.4188	0.7065	0.7764	0.9815	0.7322	0.2671
	r coefficient	−0.1332	0.0623	−0.047	−0.0038	0.0566	−0.1821

Intraocular pressure^†^	N	39	39	39	39	39	39
	*P* value	0.3603	0.5769	0.3641	0.4718	0.7215	0.7197
	r coefficient	0.1506	0.0921	−0.1494	−0.1187	−0.0589	0.0593

SBP, systolic blood pressure; DBP, diastolic blood pressure; BCVA, best-corrected visual acuity; CRT, central retinal thickness. ^†^At month 12, CRT, intraocular pressure, and acircularity index were abnormally distributed (*P* values: <0.0001, 0.0423, 0.0002, respectively, in Shapiro–Wilk's test). The Spearman correlation was used to examine correlations with other variables. Pearson correlations were analyzed for others. ^*∗*^*P* < 0.05.

**Table 6:** Correlations between the evaluated parameters and the recurrence of macular edema within 12 months.

	*P* value	OR	95% CI	
			Lower	Upper
Baseline SCP	0.909	0.988	0.800	1.220
Baseline DCP	0.827	0.968	0.721	1.298
Baseline FAZ	0.753	5.207	0.0001	153583
Baseline PERIM	0.997	1.005	0.104	9.714
Baseline AI	0.437	35.518	0.004	285773
Baseline FD-300	0.282	1.120	0.911	1.378
SCP at 6 months	0.485	0.914	0.709	1.177
DCP at 6 months	0.202	0.853	0.668	1.089
FAZ at 6 months	0.302	0.060	0.0003	12.648
PERIM at 6 months	0.424	0.596	0.167	2.123
AI at 6 months	0.423	774.743	0.000065	9.173 × 10^9^
FD-300 at 6 months	0.717	0.980	0.880	1.092
SCP at 12 months	0.229	0.483	0.637	1.114
DCP at 12 months	0.500	1.118	0.808	1.548
FAZ at 12 months	0.149	0.008	0.0000098	5.768
PERIM at 12 months	0.031^*∗*^	0.060	0.005	0.774
AI at 12 months	0.019^*∗*^	4.51 × 10^12^	131.852	1.543 × 10^23^
FD-300 at 12 months	0.227	0.885	0.709	1.103
*Changes at 12 months*				
SCP	0.954	0.942	0.126	7.037
DCP	0.249	4.089	0.373	44.775
FAZ	0.666	0.548	0.036	8.370
PERIM	0.999	1.377 × 10^8^	0	0
AI	0.398	3.731	0.177	78.802
FD-300	0.543	2.640	0.116	60.261
BCVA	0.760	0.690	0.063	7.512
CRT	0.760	0.690	0.063	7.512

SCP, superficial retinal capillary plexus; DCP, deep retinal capillary plexus; FAZ, foveal avascular zone; PERIM, perimeter; AI, acircularity index; BCVA, best-corrected visual acuity; CRT, central retinal thickness. In binary logistic regression analyses, the PERIM and AI at 12 months were significantly associated with recurrence of macular edema. ^*∗*^*P* < 0.05.

**Table 7:** Correlations between the evaluated parameters and the number of injections within 12 months.

	*P* value	OR	95% CI	
			Lower	Upper
Baseline SCP	0.909	0.988	0.800	1.220
Baseline DCP	0.827	0.968	0.721	1.298
Baseline FAZ	0.753	5.207	0.0001	153583
Baseline PERIM	0.997	1.005	0.104	9.714
Baseline AI	0.437	35.518	0.004	285773
Baseline FD-300	0.282	1.120	0.911	1.378
SCP at 6 months	0.485	0.914	0.709	1.177
DCP at 6 months	0.202	0.853	0.668	1.089
FAZ at 6 months	0.302	0.060	0.0003	12.648
PERIM at 6 months	0.424	0.596	0.167	2.123
AI at 6 months	0.423	774.743	0.000065	9.173 × 10^9^
FD-300 at 6 months	0.717	0.980	0.880	1.092
SCP at 12 months	0.229	0.483	0.637	1.114
DCP at 12 months	0.500	1.118	0.808	1.548
FAZ at 12 months	0.149	0.008	0.0000098	5.768
PERIM at 12 months	0.031^*∗*^	0.060	0.005	0.774
AI at 12 months	0.019^*∗*^	4.51 × 10^12^	131.852	1.543 × 10^23^
FD-300 at 12 months	0.227	0.885	0.709	1.103
*Changes at 12 months*				
SCP	0.954	0.942	0.126	7.037
DCP	0.249	4.089	0.373	44.775
FAZ	0.666	0.548	0.036	8.370
PERIM	0.999	1.377 × 10^8^	0	0
AI	0.398	3.731	0.177	78.802
FD-300	0.543	2.640	0.116	60.261
BCVA	0.760	0.690	0.063	7.512
CRT	0.760	0.690	0.063	7.512

SCP, superficial retinal capillary plexus; DCP, deep retinal capillary plexus; FAZ, foveal avascular zone; PERIM, perimeter; AI, acircularity index; BCVA, best-corrected visual acuity; CRT, central retinal thickness. In linear logistic regression analyses, the changes in SCP and PERIM were significantly associated with the number of injections at 12 months. ^*∗*^*P* < 0.05.

## Data Availability

The datasets used and/or analyzed during the current study are available from the corresponding author upon reasonable request.
